# Crystal structure and Hirshfeld surface analysis of 4-{2,2-di­chloro-1-[(*E*)-2-(4-methyl­phen­yl)diazen-1-yl]ethen­yl}-*N*,*N*-di­methyl­aniline

**DOI:** 10.1107/S2056989020007744

**Published:** 2020-06-19

**Authors:** Kadriye Özkaraca, Mehmet Akkurt, Namiq Q. Shikhaliyev, Ulviyya F. Askerova, Gulnar T. Suleymanova, Gunay Z. Mammadova, Sixberth Mlowe

**Affiliations:** a Institute of Natural and Applied Science, Erciyes University, 38039 Kayseri, Turkey; b Department of Physics, Faculty of Sciences, Erciyes University, 38039 Kayseri, Turkey; c Organic Chemistry Department, Baku State University, Z. Khalilov str. 23, AZ, 1148 Baku, Azerbaijan; d University of Dar es Salaam, Dar es Salaam University College of Education, Department of Chemistry, PO Box 2329, Dar es Salaam, Tanzania

**Keywords:** crystal structure, Hirshfeld surface analysis, non-covalent inter­actions, halogen and H-atom contacts

## Abstract

In the tile compound, C_17_H_17_Cl_2_N_3_, the dihedral angle between the benzene rings is 62.73 (9)°. In the crystal, there are no classical hydrogen bonds. Mol­ecules are linked by a pair of C—Cl⋯π inter­actions, forming an inversion dimer. A short inter­molecular H*L*⋯H*L* contact [Cl⋯Cl = 3.2555 (9) Å] links the dimers, forming a ribbon along the *c*-axis direction. The Hirshfeld surface analysis and two-dimensional fingerprint plots reveal that the most important contributions for the crystal packing are from H⋯H (45.4%), Cl⋯H/H⋯Cl (21.0%) and C⋯H/H⋯C (19.0%) contacts.

## Abstract 2

In the title compound, the benzene rings make a dihedral angle of 62.73 (9)° with each other. In the crystal, mol­ecules are linked by a pair of C—Cl⋯π inter­actions, forming an inversion dimer. A short H*L*⋯·H*L* contact links the dimers, forming a ribbon propagating along the *c*-axis.

## Chemical context   

Although non-covalent inter­actions are weaker than the covalent bonds, they are common and play critical roles in micellization, synthesis and catalysis as well as in forming supra­molecular structures as a result of their significant contribution to the self-assembly process (Asadov *et al.*, 2016 ▸; Maharramov *et al.*, 2010 ▸; Mahmudov *et al.*, 2019 ▸). Similar to well-explored hydrogen bonds and *π-*inter­actions (Gurbanov *et al.*, 2018 ▸; Mahmoudi *et al.*, 2018 ▸), all aspects of chemistry and physics of halogen bonding have been subject to rapidly growing inter­est over the past decade. Thus, the attachment of halogen-bond donor site(s) to organic mol­ecules can be used in the regulation of the solvatochromic, analytical, catalytic *etc*. properties of materials (Maharramov *et al.*, 2018 ▸; Mahmudov *et al.*, 2016 ▸). In a continuation of our work in this area we have functionalized the title compound, a new azo dye, which provides weak inter­molecular inter­actions of the C—Cl⋯π and C—Cl⋯Cl types.
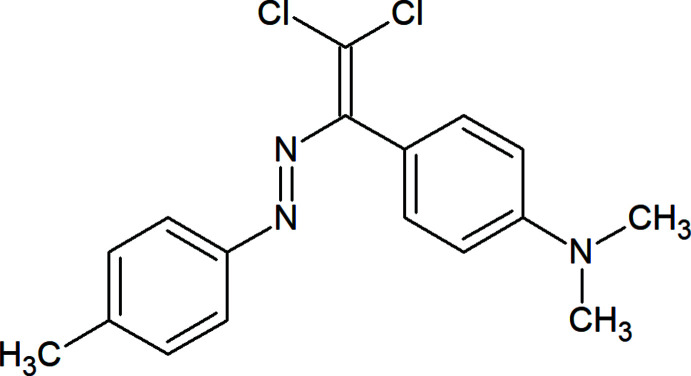


## Structural commentary   

**Figure 1:**
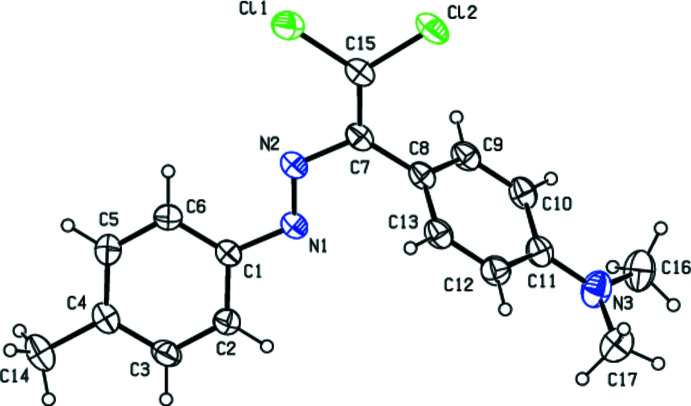
The mol­ecular structure of the title compound with displacement ellipsoids for the non-hydrogen atoms drawn at the 30% probability level.

In the title compound (Fig. 1 ▸), the dihedral angle between the C1–C6 and C8–C13 benzene rings is 62.73 (9)°. The amine N atom (N3) displaced slightly from the C8–C13 benzene ring plane, with a deviation of 0.014 (2) Å. The N1/N2/C7/C15/Cl1/Cl2 unit is approximately planar with a maximum deviation of 0.0225 (19) Å, and makes dihedral angles of 6.46 (7) and 63.06 (7)°, respectively, with the C1–C6 and C8–C11 rings.

## Supra­molecular features   

**Figure 2:**
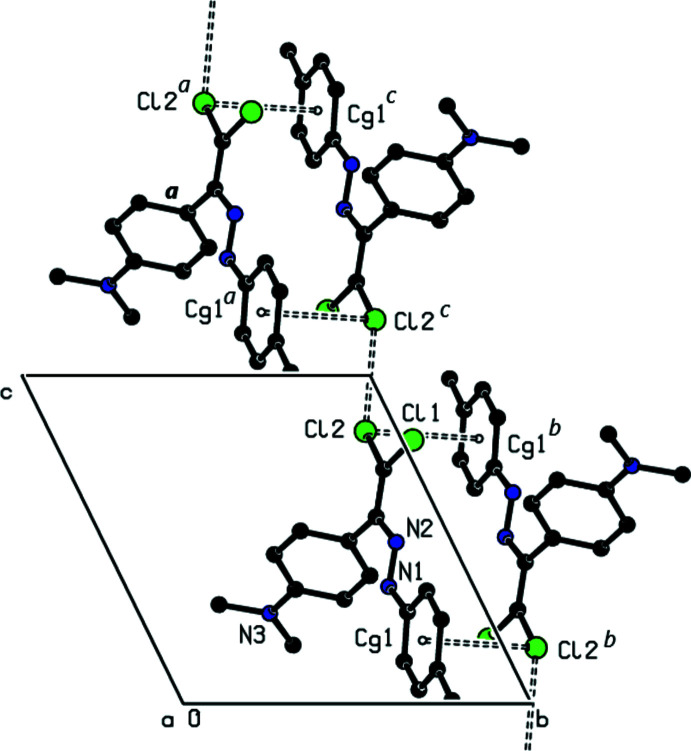
A view of the Cl⋯Cl short contacts and C—Cl⋯π inter­actions between the mol­ecules. All hydrogen atoms were omitted for clarity. [Symmetry codes: (*a*) *x*, *y*, 1 + *z*; (*b*) 1 − *x*, 2 − *y*, 1 − *z*; (*c*) 1 − *x*, 2 − *y*, 2 − *z*.]

In the crystal, there are no classical hydrogen bonds observed. Mol­ecules are linked by a pair of C—Cl⋯π inter­actions (Table 1 ▸), forming an inversion dimer. A short inter­molecular H*L*⋯H*L* contact [Cl2⋯Cl2 (1 − *x*, 2 − *y*, 2 − *z*) = 3.2555 (9) Å] links the dimers to form a ribbon along the *c*-axis direction (Figs. 2 ▸ and 3 ▸). The mol­ecular packing is further stabilized by van der Waals inter­actions between these ribbons.

**Figure 4:**
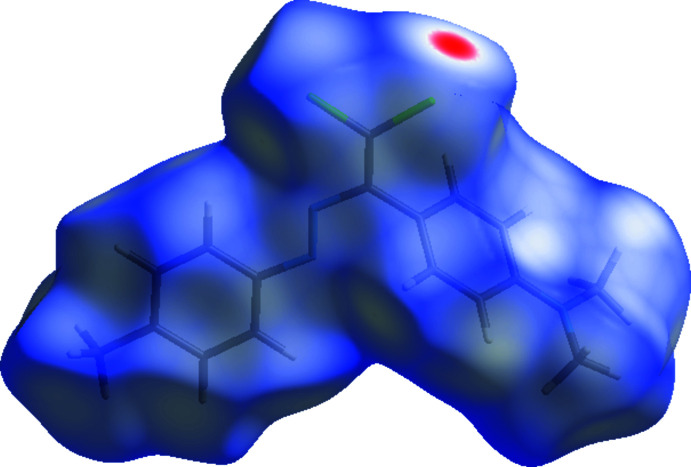
A view of the three-dimensional Hirshfeld surface for the title compound plotted over *d*_norm_ in the range −0.1388 to 1.4611 a.u.

Hirshfeld surfaces (McKinnon *et al.*, 2007 ▸) and their associated two-dimensional fingerprint plots (Spackman & McKinnon, 2002 ▸) were calculated using *CrystalExplorer17* (Turner *et al.*, 2017 ▸) to visualize the inter­molecular inter­actions in the title compound. In the Hirshfeld surface mapped over *d*_norm_ (Fig. 4 ▸), a bright-red spot near atom Cl2 indicates the short Cl⋯Cl contact. Other contacts are equal to or longer than the sum of van der Walls radii.

**Figure 5:**
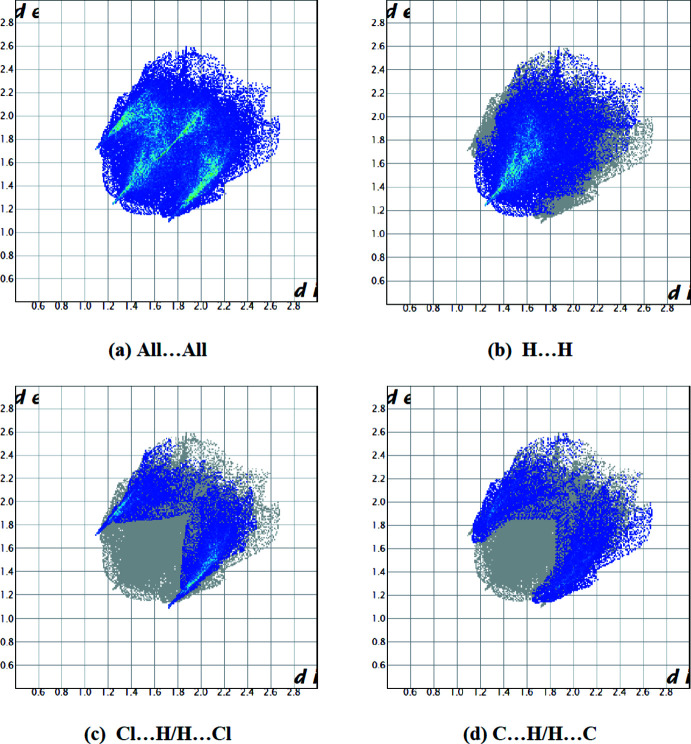
Two-dimensional fingerprint plots for the title compound, showing (*a*) all inter­actions, and delineated into (*b*) H⋯H, (*c*) Cl⋯H/H⋯Cl and (*d*) C⋯H/H⋯C inter­actions. The *d*_i_ and *d*_e_ values are the closest inter­nal and external distances (in Å) from given points on the Hirshfeld surface.

The overall two-dimensional fingerprint plot and those delineated into H⋯H, Cl⋯H/H⋯Cl and C⋯H/H⋯C contacts (McKinnon *et al.*, 2007 ▸) are illustrated in Fig. 5 ▸. The most important inter­action is H⋯H, contributing 45.4% to the overall crystal packing (Fig. 5 ▸ *b*), which is reflected as widely scattered points of high density due to the large hydrogen content of the mol­ecule with the tip at *d*_e_ = *d*_i_ = 1.25 Å. The Cl⋯H/H⋯Cl inter­actions appear as two symmetrical broad wings with *d*_e_ + *d*_i_ ≃ 2.80 Å and contribute 21.0% to the Hirshfeld surface (Fig. 5 ▸ *c*). The pair of characteristic wings in the fingerprint plot delineated into H⋯C/C⋯H contacts (Fig. 5 ▸ *d*; 19.0% contribution) have the tips at *d*_e_ + *d*_i_ ≃ 2.80 Å. The remaining contributions are from N⋯H/H⋯N (5.9%), Cl⋯C/C⋯Cl (3.8%), Cl⋯Cl (1.5%), C⋯C (1.5%), N⋯C/C⋯N (1.1%), N⋯Cl/Cl⋯N (0.5%) and N⋯N (0.4%) contacts, which have a negligible effect on the packing.

## Database survey   

The title compound is similar to 4-{2,2-di­chloro-1-[(*E*)-(4-fluoro­phen­yl) diazen­yl]ethen­yl}-*N*,*N*-di­methyl­aniline (CSD refcode DULTAI; Özkaraca *et al.*, 2020 ▸), and closely resembles four other compounds, *viz.* 1-(4-bromo­phen­yl)-2-[2,2-di­chloro-1-(4-nitro­phen­yl)ethen­yl]diazene (HONBOE; Akkurt *et al.*, 2019 ▸), 1-(4-chloro­phen­yl)-2-[2,2-di­chloro-1-(4-nitro­phen­yl)ethen­yl]diazene (HONBUK; Akkurt *et al.*, 2019 ▸), 1-(4-chloro­phen­yl)-2-[2,2-di­chloro-1-(4-fluoro­phen­yl)ethen­yl]diazene (HODQAV; Shikhaliyev *et al.*, 2019 ▸) and 1-[2,2-di­chloro-1-(4-nitro­phen­yl)ethen­yl]-2-(4-fluoro­phen­yl)diazene (XIZREG; Atioğlu *et al.*, 2019 ▸).

The crystal structure of DULTAI is stabilized by C—Cl⋯π and van der Waals inter­actions. In the crystals of HONBOE and HONBUK, mol­ecules are linked through weak *X*⋯Cl contacts (*X* = Br for HONBOE and Cl for HONBUK), C—H⋯Cl and C—Cl⋯π inter­actions into sheets parallel to the *ab* plane. In HODQAV, mol­ecules are stacked in columns along the *a* axis *via* weak C—H⋯Cl hydrogen bonds and face-to-face π–π stacking inter­actions. The crystal packing is further stabilized by short Cl⋯Cl contacts. In XIZREG, mol­ecules are linked by C—H⋯O hydrogen bonds into zigzag chains running along the *c*-axis direction. The crystal packing is further stabilized by C—Cl⋯π, C—F⋯π and N—O⋯π inter­actions.

## Synthesis and crystallization   

The title dye compound was synthesized according to the reported method (Atioğlu *et al.*, 2019 ▸). A 20 mL screw neck vial was charged with DMSO (10 mL), (*Z*)-*N*,*N*-di­meth­yl-4-{[2-(*p*-tol­yl)hydrazineyl­idene]meth­yl}aniline (253 mg, 1 mmol), tetra­methyl­ethylenedi­amine (TMEDA) (295 mg, 2.5 mmol), CuCl (2 mg, 0.02 mmol) and CCl_4_ (20 mmol, 10 equiv). After 1–3 h (when TLC analysis showed complete consumption of corresponding Schiff base), the reaction mixture was poured into 100 mL of dilute HCl (∼0.01 *M*, pH = 2–3), and extracted with di­chloro­methane (3 × 20 mL). The combined organic phase was washed with water (3 × 50 mL), brine (30 mL), dried over anhydrous Na_2_SO_4_ and concentrated *in vacuo* using a rotary evaporator. The residue was purified by column chromatography on silica gel using appropriate mixtures of hexane and di­chloro­methane (3:1–1:1). Single crystals suitable for X-ray analysis were obtained by slow evaporation of an ethanol solution. Orange solid (79%); m.p. 386 K. Analysis calculated for C_17_H_17_Cl_2_N_3_ (*M* = 334.24): C 61.09, H 5.13, N 12.57; found: C 61.03, H 5.07, N 12.53%. ^1^H NMR (300 MHz, CDCl_3_) *δ* 2.34 (3H, ArMe), 3.06 (6H, NMe_2_), 6.80–7.79 (8H, Ar). ^13^C NMR (75MHz, CDCl_3_) δ 152.33, 151.30, 150.28, 142.01, 133.28, 131.15, 129.71, 123.30, 119.47, 111.42, 40.30, 21.62. ESI–MS: *m*/*z*: 335.22 [*M* + H]^+^.

## Refinement details   

Crystal data, data collection and structure refinement details are summarized in Table 2 ▸. The C-bound H atoms were positioned geometrically (C—H = 0.93–0.96 Å) and refined as riding with *U*_iso_(H) = 1.5 or 1.2*U*_eq_(C).

## Supplementary Material

Crystal structure: contains datablock(s) general, I. DOI: 10.1107/S2056989020007744/is5539sup1.cif

Structure factors: contains datablock(s) I. DOI: 10.1107/S2056989020007744/is5539Isup2.hkl

Supporting information file. DOI: 10.1107/S2056989020007744/is5539Isup3.cml

CCDC reference: 2008423

Additional supporting information:  crystallographic information; 3D view; checkCIF report

## Figures and Tables

**Figure 3:**
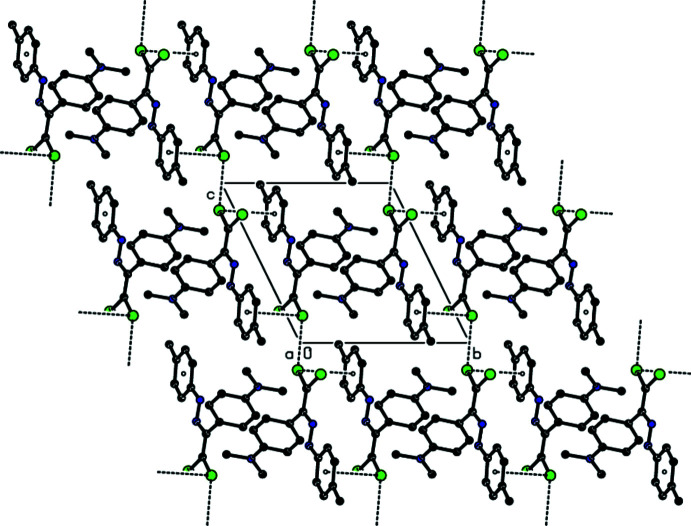
A packing diagram of the title compound, viewed along the *a*-axis direction. All hydrogen atoms were omitted for clarity.

**Table 1:** Hydrogen-bond geometry (Å, °) *Cg*1 is the centroid of the C1–C6 benzene ring.

*D*—H⋯*A*	*D*—H	H⋯*A*	*D*⋯*A*	*D*—H⋯*A*
C15—Cl2⋯*Cg*1^i^	1.71 (1)	3.60 (1)	4.065 (2)	93 (1)

Symmetry code: (i) 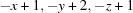.

**Table 2:** Experimental details

Crystal data	
Chemical formula	C_17_H_17_Cl_2_N_3_
*M*_r_	334.23
Crystal system, space group	Triclinic, *P* 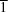
Temperature (K)	296
*a*, *b*, *c* (Å)	9.5967 (15), 9.6767 (15), 10.8043 (17)
α, β, γ (°)	114.162 (5), 109.930 (5), 90.917 (6)
*V* (Å^3^)	846.3 (2)
*Z*	2
Radiation type	Mo *K*α
μ (mm^−1^)	0.38
Crystal size (mm)	0.34 × 0.31 × 0.25

Data collection	
Diffractometer	Bruker APEXII PHOTON 100 detector
Absorption correction	Multi-scan (*SADABS*; Bruker, 2003 ▸)
*T*_min_, *T*_max_	0.875, 0.894
No. of measured, independent and observed [*I* > 2σ(*I*)] reflections	13168, 3286, 2808
*R*_int_	0.036
(sin θ/λ)_max_ (Å^−1^)	0.620

Refinement	
*R*[*F* ^2^ > 2σ(*F* ^2^)], *wR*(*F* ^2^), *S*	0.041, 0.109, 1.05
No. of reflections	3286
No. of parameters	202
H-atom treatment	H-atom parameters constrained
Δρ_max_, Δρ_min_ (e Å^−3^)	0.19, −0.30

Computer programs: *APEX3* and *SAINT* (Bruker, 2007 ▸), *SHELXT2016/6* (Sheldrick, 2015*a* ▸), *SHELXL2016/6* (Sheldrick, 2015*b* ▸), *ORTEP-3 for Windows* (Farrugia, 2012 ▸) and *PLATON* (Spek, 2020 ▸).

## supplementary crystallographic information

### Crystal data

**Table:**

C_17_H_17_Cl_2_N_3_	*Z* = 2
*M**_r_* = 334.23	*F*(000) = 348
Triclinic, *P*1	*D*_x_ = 1.312 Mg m^−^^3^
*a* = 9.5967 (15) Å	Mo *K*α radiation, λ = 0.71073 Å
*b* = 9.6767 (15) Å	Cell parameters from 8175 reflections
*c* = 10.8043 (17) Å	θ = 2.2–26.1°
α = 114.162 (5)°	µ = 0.38 mm^−^^1^
β = 109.930 (5)°	*T* = 296 K
γ = 90.917 (6)°	Block, orange
*V* = 846.3 (2) Å^3^	0.34 × 0.31 × 0.25 mm

### Data collection

**Table:**

Bruker APEXII PHOTON 100 detector diffractometer	2808 reflections with *I* > 2σ(*I*)
φ and ω scans	*R*_int_ = 0.036
Absorption correction: multi-scan (SADABS; Bruker, 2003)	θ_max_ = 26.1°, θ_min_ = 2.4°
*T*_min_ = 0.875, *T*_max_ = 0.894	*h* = −11→11
13168 measured reflections	*k* = −11→11
3286 independent reflections	*l* = −13→13

### Refinement

**Table:**

Refinement on *F*^2^	0 restraints
Least-squares matrix: full	Hydrogen site location: inferred from neighbouring sites
*R*[*F*^2^ > 2σ(*F*^2^)] = 0.041	H-atom parameters constrained
*wR*(*F*^2^) = 0.109	*w* = 1/[σ^2^(*F*_o_^2^) + (0.0413*P*)^2^ + 0.2744*P*] where *P* = (*F*_o_^2^ + 2*F*_c_^2^)/3
*S* = 1.05	(Δ/σ)_max_ = 0.001
3286 reflections	Δρ_max_ = 0.19 e Å^−^^3^
202 parameters	Δρ_min_ = −0.30 e Å^−^^3^

### Special details

**Table:**

Geometry. All esds (except the esd in the dihedral angle between two l.s. planes) are estimated using the full covariance matrix. The cell esds are taken into account individually in the estimation of esds in distances, angles and torsion angles; correlations between esds in cell parameters are only used when they are defined by crystal symmetry. An approximate (isotropic) treatment of cell esds is used for estimating esds involving l.s. planes.

### Fractional atomic coordinates and isotropic or equivalent isotropic displacement parameters (Å^2^)

**Table:**

	*x*	*y*	*z*	*U*_iso_*/*U*_eq_	
C1	0.62327 (18)	0.77347 (17)	0.27963 (16)	0.0430 (3)	
C2	0.55622 (19)	0.69400 (19)	0.12860 (18)	0.0511 (4)	
H2A	0.458599	0.638289	0.084781	0.061*	
C3	0.6330 (2)	0.6968 (2)	0.04271 (18)	0.0556 (4)	
H3A	0.586196	0.643088	−0.058810	0.067*	
C4	0.7787 (2)	0.77812 (19)	0.1045 (2)	0.0524 (4)	
C5	0.8437 (2)	0.8594 (2)	0.2560 (2)	0.0546 (4)	
H5A	0.940803	0.916211	0.299537	0.065*	
C6	0.76843 (19)	0.85845 (19)	0.34383 (18)	0.0504 (4)	
H6A	0.814274	0.914147	0.445239	0.060*	
C7	0.50704 (19)	0.81792 (18)	0.56909 (17)	0.0466 (4)	
C8	0.36451 (18)	0.70537 (18)	0.49547 (17)	0.0459 (4)	
C9	0.3511 (2)	0.58051 (19)	0.52632 (19)	0.0516 (4)	
H9A	0.432363	0.569863	0.597144	0.062*	
C10	0.2204 (2)	0.4724 (2)	0.4546 (2)	0.0533 (4)	
H10A	0.215848	0.390240	0.477657	0.064*	
C11	0.09506 (19)	0.48342 (19)	0.34826 (19)	0.0508 (4)	
C12	0.1089 (2)	0.6101 (2)	0.3183 (2)	0.0553 (4)	
H12A	0.027551	0.622051	0.248519	0.066*	
C13	0.2401 (2)	0.7167 (2)	0.39000 (19)	0.0527 (4)	
H13A	0.245451	0.798834	0.366953	0.063*	
C14	0.8640 (3)	0.7763 (3)	0.0098 (3)	0.0783 (6)	
H14A	0.889625	0.879225	0.023489	0.118*	
H14B	0.802118	0.712384	−0.091347	0.118*	
H14C	0.954396	0.736056	0.036936	0.118*	
C15	0.5678 (2)	0.9067 (2)	0.71430 (18)	0.0517 (4)	
C16	−0.0462 (3)	0.2427 (2)	0.3018 (3)	0.0794 (6)	
H16A	0.034913	0.189498	0.288174	0.119*	
H16B	−0.140759	0.175108	0.234394	0.119*	
H16C	−0.039713	0.274646	0.400506	0.119*	
C17	−0.1688 (2)	0.3981 (3)	0.1783 (3)	0.0790 (6)	
H17A	−0.149409	0.401936	0.098146	0.118*	
H17B	−0.196497	0.493105	0.230865	0.118*	
H17C	−0.249623	0.314219	0.140945	0.118*	
Cl1	0.73773 (6)	1.03097 (6)	0.80319 (5)	0.07071 (18)	
Cl2	0.48299 (7)	0.90774 (6)	0.82988 (5)	0.07116 (18)	
N1	0.53535 (15)	0.75555 (15)	0.35683 (14)	0.0473 (3)	
N2	0.59263 (16)	0.83959 (15)	0.49225 (14)	0.0477 (3)	
N3	−0.03590 (19)	0.37582 (19)	0.2751 (2)	0.0710 (5)	

### Atomic displacement parameters (Å^2^)

**Table:**

	*U*^11^	*U*^22^	*U*^33^	*U*^12^	*U*^13^	*U*^23^
C1	0.0496 (8)	0.0432 (8)	0.0417 (7)	0.0113 (6)	0.0216 (6)	0.0203 (6)
C2	0.0529 (9)	0.0533 (9)	0.0430 (8)	0.0018 (7)	0.0187 (7)	0.0176 (7)
C3	0.0691 (11)	0.0580 (10)	0.0405 (8)	0.0085 (8)	0.0239 (8)	0.0201 (7)
C4	0.0623 (10)	0.0522 (9)	0.0586 (10)	0.0173 (8)	0.0346 (8)	0.0295 (8)
C5	0.0500 (9)	0.0553 (9)	0.0615 (10)	0.0073 (7)	0.0249 (8)	0.0258 (8)
C6	0.0524 (9)	0.0511 (9)	0.0422 (8)	0.0061 (7)	0.0166 (7)	0.0168 (7)
C7	0.0562 (9)	0.0502 (8)	0.0468 (8)	0.0194 (7)	0.0268 (7)	0.0277 (7)
C8	0.0526 (9)	0.0510 (8)	0.0468 (8)	0.0180 (7)	0.0279 (7)	0.0259 (7)
C9	0.0584 (10)	0.0587 (10)	0.0550 (9)	0.0246 (8)	0.0286 (8)	0.0349 (8)
C10	0.0627 (10)	0.0520 (9)	0.0665 (10)	0.0217 (8)	0.0348 (9)	0.0372 (8)
C11	0.0547 (9)	0.0504 (9)	0.0585 (9)	0.0183 (7)	0.0311 (8)	0.0264 (8)
C12	0.0539 (10)	0.0627 (10)	0.0604 (10)	0.0189 (8)	0.0223 (8)	0.0367 (9)
C13	0.0615 (10)	0.0551 (9)	0.0593 (10)	0.0200 (8)	0.0283 (8)	0.0369 (8)
C14	0.0881 (15)	0.0940 (15)	0.0805 (14)	0.0209 (12)	0.0559 (13)	0.0447 (12)
C15	0.0640 (10)	0.0548 (9)	0.0462 (8)	0.0167 (8)	0.0275 (8)	0.0259 (7)
C16	0.0795 (14)	0.0573 (11)	0.1090 (18)	0.0117 (10)	0.0443 (13)	0.0369 (12)
C17	0.0613 (12)	0.0817 (14)	0.0822 (14)	0.0056 (10)	0.0186 (11)	0.0327 (12)
Cl1	0.0783 (3)	0.0738 (3)	0.0505 (3)	−0.0015 (2)	0.0190 (2)	0.0236 (2)
Cl2	0.0960 (4)	0.0798 (3)	0.0523 (3)	0.0182 (3)	0.0452 (3)	0.0289 (2)
N1	0.0530 (8)	0.0512 (7)	0.0426 (7)	0.0111 (6)	0.0231 (6)	0.0212 (6)
N2	0.0563 (8)	0.0500 (7)	0.0440 (7)	0.0118 (6)	0.0248 (6)	0.0228 (6)
N3	0.0598 (9)	0.0643 (10)	0.0933 (12)	0.0091 (7)	0.0258 (9)	0.0415 (9)

### Geometric parameters (Å, º)

**Table:**

C1—C2	1.382 (2)	C10—H10A	0.9300
C1—C6	1.391 (2)	C11—N3	1.377 (2)
C1—N1	1.4251 (19)	C11—C12	1.405 (2)
C2—C3	1.375 (2)	C12—C13	1.375 (3)
C2—H2A	0.9300	C12—H12A	0.9300
C3—C4	1.385 (3)	C13—H13A	0.9300
C3—H3A	0.9300	C14—H14A	0.9600
C4—C5	1.386 (3)	C14—H14B	0.9600
C4—C14	1.507 (2)	C14—H14C	0.9600
C5—C6	1.377 (2)	C15—Cl2	1.7046 (17)
C5—H5A	0.9300	C15—Cl1	1.7179 (19)
C6—H6A	0.9300	C16—N3	1.439 (3)
C7—C15	1.340 (2)	C16—H16A	0.9600
C7—N2	1.418 (2)	C16—H16B	0.9600
C7—C8	1.480 (2)	C16—H16C	0.9600
C8—C13	1.384 (2)	C17—N3	1.432 (3)
C8—C9	1.393 (2)	C17—H17A	0.9600
C9—C10	1.378 (3)	C17—H17B	0.9600
C9—H9A	0.9300	C17—H17C	0.9600
C10—C11	1.393 (2)	N1—N2	1.2520 (18)
			
C2—C1—C6	119.28 (14)	C13—C12—C11	121.43 (16)
C2—C1—N1	115.14 (14)	C13—C12—H12A	119.3
C6—C1—N1	125.54 (14)	C11—C12—H12A	119.3
C3—C2—C1	120.38 (16)	C12—C13—C8	121.84 (15)
C3—C2—H2A	119.8	C12—C13—H13A	119.1
C1—C2—H2A	119.8	C8—C13—H13A	119.1
C2—C3—C4	121.27 (16)	C4—C14—H14A	109.5
C2—C3—H3A	119.4	C4—C14—H14B	109.5
C4—C3—H3A	119.4	H14A—C14—H14B	109.5
C3—C4—C5	117.74 (15)	C4—C14—H14C	109.5
C3—C4—C14	120.93 (17)	H14A—C14—H14C	109.5
C5—C4—C14	121.33 (18)	H14B—C14—H14C	109.5
C6—C5—C4	121.83 (16)	C7—C15—Cl2	123.03 (14)
C6—C5—H5A	119.1	C7—C15—Cl1	123.91 (13)
C4—C5—H5A	119.1	Cl2—C15—Cl1	113.06 (10)
C5—C6—C1	119.47 (15)	N3—C16—H16A	109.5
C5—C6—H6A	120.3	N3—C16—H16B	109.5
C1—C6—H6A	120.3	H16A—C16—H16B	109.5
C15—C7—N2	114.01 (15)	N3—C16—H16C	109.5
C15—C7—C8	123.11 (15)	H16A—C16—H16C	109.5
N2—C7—C8	122.87 (14)	H16B—C16—H16C	109.5
C13—C8—C9	116.98 (16)	N3—C17—H17A	109.5
C13—C8—C7	121.61 (14)	N3—C17—H17B	109.5
C9—C8—C7	121.38 (15)	H17A—C17—H17B	109.5
C10—C9—C8	121.69 (16)	N3—C17—H17C	109.5
C10—C9—H9A	119.2	H17A—C17—H17C	109.5
C8—C9—H9A	119.2	H17B—C17—H17C	109.5
C9—C10—C11	121.48 (15)	N2—N1—C1	113.76 (13)
C9—C10—H10A	119.3	N1—N2—C7	113.53 (14)
C11—C10—H10A	119.3	C11—N3—C17	121.17 (17)
N3—C11—C10	122.12 (15)	C11—N3—C16	120.75 (18)
N3—C11—C12	121.30 (16)	C17—N3—C16	117.94 (18)
C10—C11—C12	116.58 (16)		
			
C6—C1—C2—C3	1.0 (2)	N3—C11—C12—C13	−179.23 (17)
N1—C1—C2—C3	−177.02 (15)	C10—C11—C12—C13	0.4 (3)
C1—C2—C3—C4	0.3 (3)	C11—C12—C13—C8	−0.4 (3)
C2—C3—C4—C5	−1.3 (3)	C9—C8—C13—C12	−0.1 (2)
C2—C3—C4—C14	177.94 (17)	C7—C8—C13—C12	178.13 (15)
C3—C4—C5—C6	1.1 (3)	N2—C7—C15—Cl2	177.33 (11)
C14—C4—C5—C6	−178.15 (17)	C8—C7—C15—Cl2	−3.6 (2)
C4—C5—C6—C1	0.2 (3)	N2—C7—C15—Cl1	−2.5 (2)
C2—C1—C6—C5	−1.3 (2)	C8—C7—C15—Cl1	176.56 (12)
N1—C1—C6—C5	176.59 (15)	C2—C1—N1—N2	−173.79 (14)
C15—C7—C8—C13	119.31 (18)	C6—C1—N1—N2	8.3 (2)
N2—C7—C8—C13	−61.7 (2)	C1—N1—N2—C7	−178.18 (12)
C15—C7—C8—C9	−62.6 (2)	C15—C7—N2—N1	179.64 (14)
N2—C7—C8—C9	116.43 (17)	C8—C7—N2—N1	0.6 (2)
C13—C8—C9—C10	0.4 (2)	C10—C11—N3—C17	172.96 (19)
C7—C8—C9—C10	−177.74 (15)	C12—C11—N3—C17	−7.4 (3)
C8—C9—C10—C11	−0.4 (3)	C10—C11—N3—C16	−2.6 (3)
C9—C10—C11—N3	179.61 (16)	C12—C11—N3—C16	177.03 (18)
C9—C10—C11—C12	0.0 (2)		

### Hydrogen-bond geometry (Å, º)

*Cg*1 is the centroid of the C1–C6 benzene ring.

**Table:**

*D*—H···*A*	*D*—H	H···*A*	*D*···*A*	*D*—H···*A*
C15—Cl2···*Cg*1^i^	1.71 (1)	3.60 (1)	4.065 (2)	93 (1)

Symmetry code: (i) −*x*+1, −*y*+2, −*z*+1.
